# A Novel Sensor Data Pre-Processing Methodology for the Internet of Things Using Anomaly Detection and Transfer-By-Subspace-Similarity Transformation

**DOI:** 10.3390/s19204536

**Published:** 2019-10-18

**Authors:** Yan Zhong, Simon Fong, Shimin Hu, Raymond Wong, Weiwei Lin

**Affiliations:** 1 Department of Big Data and Cloud Computing, Zhuhai Institutes of Advanced Technology of the Chinese Academy of Sciences, Zhuhai 519000, China; mb75500@um.edu.mo; 2 Department of Computer and Information Science, University of Macau, Taipa 999078, Macau; yb77401@um.edu.mo; 3 School of Computer Science & Engineering, University of New South Wales, Sydney 2052, Australia; wong@cse.unsw.edu.au; 4 School of Computer Science and Engineering, South China University of Technology, Guangzhou 510006, China; linww@scut.edu.cn

**Keywords:** Internet of Things, sensor data, preprocessing, subspace similarity

## Abstract

The Internet of Things (IoT) and sensors are becoming increasingly popular, especially in monitoring large and ambient environments. Applications that embrace IoT and sensors often require mining the data feeds that are collected at frequent intervals for intelligence. Despite the fact that such sensor data are massive, most of the data contents are identical and repetitive; for example, human traffic in a park at night. Most of the traditional classification algorithms were originally formulated decades ago, and they were not designed to handle such sensor data effectively. Hence, the performance of the learned model is often poor because of the small granularity in classification and the sporadic patterns in the data. To improve the quality of data mining from the IoT data, a new pre-processing methodology based on subspace similarity detection is proposed. Our method can be well integrated with traditional data mining algorithms and anomaly detection methods. The pre-processing method is flexible for handling similar kinds of sensor data that are sporadic in nature that exist in many ambient sensing applications. The proposed methodology is evaluated by extensive experiment with a collection of classical data mining models. An improvement over the precision rate is shown by using the proposed method.

## 1. Introduction

The infrastructure of the Internet of Things (IoT) is establishing rapidly recently, with the hype of smart cities over the world. Many ambient-sensing applications subsequently were developed that tap into the maturity of IoT technology [1]. From these applications and the proliferation of sensor equipment and ubiquitous communication technologies, we are able to collect a huge amount of useful data, which was not possible before [2,3].

**Figure 1:**
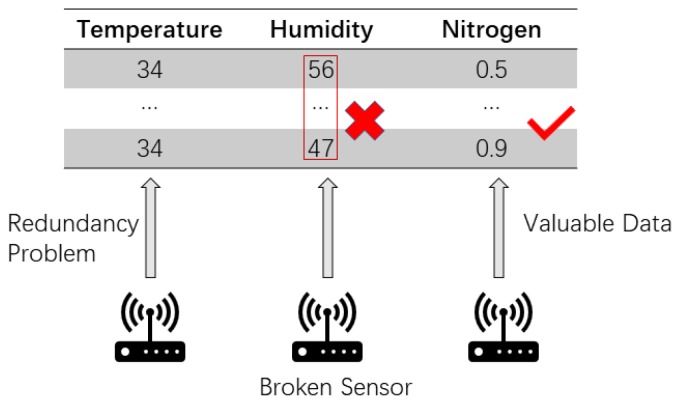
Kinds of problem in the Internet of Things.

As the Figure 1 shows, there are some problem in the internet of things.For example, a typical and challenging ambient sensing application namely human activity recognition (HAR) collects data about human activities, such as walking, running and standing, in a confined space [4]. HAR tries to make sense of a massive amount data that is collected continuously for analyzing what a person is doing over a long period of time. Another typical application of ambient sensing focuses on detecting unusual environmental occurrences by using many outdoor sensors such as atmospheric sensors [5]. Although sensors have become ubiquitous and their applications are prevalent covering every aspect of our life, the data mining component in an IoT system has to keep up its effectiveness with the sheer volume of dynamic data. Sensor data are data feeds which exhibit some patterns when they are zoomed out and viewed longitudinally. The patterns are somewhat different from the structured datasets that are used traditionally for supervised learning. Earlier on, some researchers advocated that this kind of sensor data has unique characteristics, and it is unsuitable for use directly in data analysis [6]. First, the sensor data collected by the sensor are usually in numerical values. Second, the frequency of collection is relatively quick, and a lot of data can be collected within a few seconds at a time, depending on the sampling rate. Third, the adjacent data cells along a sensor data sequence may be very similar to each other without any significant changes in values over a period of time. For instance, a sensor that is tasked to monitor the activity of a sleeping baby or a sitting phone operator: their body postures change relatively little in slow or still motions; the same goes for monitoring the humidity of a forest or agriculture in fine and stable weather [7]. Fourth, sensor data may be corrupted by noise and losing useful information due to various transmission errors or malfunctioning sensors [8]. It is not uncommon for sensors that collect bogus data due to unreliable medium or external interference in a large scale IoT network deployed in harsh environment.

From the perspective of data mining, the process may not know whether a piece of data is noisy or a plain outlier, until and unless the fault is pinpointed. For example, when a sensor malfunctions, it sends incorrect readings to the server. The wrong data will not be discovered though they are more than useless for training a data mining model. As a result, the induced model is degraded by junk data. The wrong prediction outputted from the data mining model would propagate to throughout the IoT network and eventually to the final [9].

Basically, the characteristic of the IoT data collected by sensors are sequential and huge. The data values are largely repetitive and noises that have irregular values may easily go unnoticed in the data. Many users would aggregate the data for deriving descriptive statistics such as mean and distributions. Relatively they pay less attention to individual pieces of data at the micro-scale. It is known that sensor data are often generated at narrow intervals at the high sampling rate. Some problematic data that have gone unnoticed will cause the classifiers drop in performance during operation. At the data level, it is difficult for the model induction process to know whether an incoming training data will be counter-productive to the supervised learning. This may require post-processing or feedback-learning. A simpler method is pre-processing, which is usually fast and lightweight suitable for dynamic IoT scenarios.

Some popular pre-processing methods, such as Principle Component Analysis (PCA) [10] only change the original data by reducing the attribute dimension. Dimension reduction may not be so effective here because sensor data may have only a few attributes about the sensor readings. Sensor data are time-series that come as training data feed in sequential manner. Some regularization methods [11] only change the scope of the data, and they cannot effectively isolate outliers from a data feed of mediocre values. Changing the time interval of the instance by using a simple sliding window and statistical methods is possible and straightforward, but it could be further improved. Some efficient and effective pre-processing mechanism is required for upholding the performance of a trained model. For solving the problem of “repetitive and redundant data” in an IoT situation, a novel pre-processing methodology is proposed in this article. In this paper, the proposed pre-processing part of a classification model calculates the probability between the subspace of source sequential data and the target data. This model transforms the subsequent input data into probability data in a period by the length of sliding windows that controls the time interval hence the resolution. It focuses on the data features while maintaining the data original structures.

The contributions of this paper are as follows: (i) a pre-processing method suitable for sensor data that may have persistent and redundant data values is proposed. The method converts the original data values into probabilities that are computed based on the similarity of subspace; (ii) The pre-processing method could set the size of the subspace and the length of the sliding window, and it can effectively combine the needs of the time segment analysis in the real task. (iii)The advantages of proposed pre-processing mechanisms can combine perfectly with different models, reducing the sensitivity to noisy data and redundancy problems. The precision of the classification model will be improved by using this pre-processing method, rather than directly using classification algorithms alone. The source code of this new pre-processing methodology can be downloaded for testing and verification at https://github.com/Ayo616/TBSS.

In Section 2, some related work about the pre-processing methods that are applicable for sensor data are introduced. In Section 3, the proposed pre-processing methodology is explained in detail. In Section 4, validation experiment is designed for evaluating the performance of our method. Empirical datasets are used in the comparison of other methods. In Section 5, the work is concluded.

## 2. Literature Review

With the advancement of communication technologies and, electronic sensing devices are increasing, sensor-centric IoT technologies and applications experienced rapid growth. From recent statistics [12], by 2017 the total value of the IoT industry reached 29 billion U.S. dollars. This huge market has attracted attention from both practitioners and academic researchers.

Over the years, some companies have been developing and building IoT smart systems such as smart homes, smart transportation, and smart security [13,14,15]. These technologies are aimed at solving the specific problem for a relatively pure dataset in Internet of Things.

The massive deployment of IoT devices helps people to obtain large amounts of sensory data. How to tap valuable information from this vast amount of data and form knowledge to serve life more effectively is an important issue [16]. Some researchers have tried to use current data mining technology in the development of the Internet of Things to make the Internet of Things more intelligent [17,18,19,20]. These methods under the IoT framework could usefully solve some parts of the RFID mining task problem.

Clustering is commonly used in data mining of the Internet of Things. The most common clustering method is K-means [21]. K-means is very mature in traditional data mining. It divides a dataset into several clusters. K-means have been widely used in unsupervised classification task. The distribution of Internet of Things data in some cases is a clustering problem [22,23], but the classification results presented by clustering are only similar data and cannot be judged. If people are unfamiliar or unclear with the collected data, they cannot rely on the clustering results to precisely dig out effective knowledge.

In supervised learning, people often use decision tree algorithms for data mining of the Internet of Things [24,25]. In addition, probabilistic models are also widely used, such as the Naive Bayesian model [26,27]. In machine learning, there is also a simple and efficient classification method that is SVM [28], combining with the kernel function can linearly separate data in high-dimensional space. SVM methods are suitable for small dataset due to its long learning time. Finding a hyperplane in SVM is time-consuming because it iteratively tries and searches for the most appropriate non-linear division among the data.

However, the traditional classification model shows an unstable performance on the actual sensor data. That is because of the unique nature of the Internet of Things data. Through analysis and observation, all collected sensor data have highly repetitive characteristics and often contain noisy data. The source of these noise data may be due to sensor detection errors. That leads to too many samples of negative instances in the classification process, and the accuracy of the training model will decrease. In addition, in real life, people pay more attention to the results of a period, which is inconsistent with the phenomenon of collecting data in seconds.

Some research [10] used pre-processing of the dimension to deal with the sensor data problem. It is advantageous for tackling the dimension explosion problem. However, the useless features will increase the amount of calculation and it is not beneficial for the model’s precision. So the performance of these methods is not very good in sensor data mining because this type of methods only affects the feature of instance [11]. Sensor data have a time-series feature, so some research uses sliding windows as pre-processing methodology to adjust the time interval and do statistical computation within the windows. This way is straightforward and easy to implement but still affected by noisy data. Its contribution to the performance of the model is limited [29,30] because it depends on the data purity and special requirement of task.

Our intuition for this method is that a subspace is corresponding to a behavior state. No matter how the redundant in data, the corresponding space is limited. We could detection the similarity of space to identify the status. Therefore, we propose a new type of data pre-processing methodology called Transfer by Subspace Similarity (TBSS). TBSS constructs subspace from the initial dataset. Our proposed TBSS methodology combines an anomaly detection algorithm that derives a probability table, and finally using just a traditional classification method for classification. TBSS pre-processing method can significantly improve the precision of classification. Moreover, this pre-processing methodology can be combined with various classification methods and anomaly detection methods, as well as it is flexible for real-time activity detection.

## 3. Methodology

### 3.1. Problem Definitions

Suppose we have a training dataset $D_{train}$, $d_{i}^{j}=\left(x_{i}^{j},c_{n}\right),d\in D_{train}$, it includes original sequence training data. These data are all from the sensors.

The training dataset includes different labels. The collection of these labels is $C=\left\{c_{1},c_{2},\ldots ,c_{n}\right\}$, $c_{n}\in C$. These labels indicates the the status of object at a moment.

Each instance in dataset has a set of features. $x_{i}=\left\{x_{i}^{1},x_{i}^{2},\cdots x_{i}^{j}\right\}$, $x_{i}\in X$. The collection of these features without label is $X=\left\{x_{1},x_{2},\ldots ,x_{i}\right\}$.

Our task is to design an algorithm to deal with these sequence data in order to improve the data mining quality in the Internet of things. So we need to transfer the dataset *D* to the $\hat{D}$, $\hat{d}=\left(x_{e}^{n},c_{n}\right),\hat{d}\in \hat{D}$.

$$D\overset{TBSS}{\to }\hat{D}$$

### 3.2. Reconstruct Training Data Table

In this step, we deal with the original training data table. We could divide some group from the original dataset according to the different labels. We get collection τ based on different class.

(1) $$\tau ={\left\{T_{0}^{C_{m}}\right\}}_{m=1}^{n}$$

Next we construct a sub-dataset Δ from the τ and the sample size of the Δ is $z_{\Delta }$. The τ represent the datum of a space.

(2) $$\Delta ={\left\{ST_{0}^{C_{m}}\right\}}_{m=1}^{n}$$

We construct a sub-dataset Γ from the τ,and the sample size of Γ is $z_{\Gamma }$. The Γ is used to compare with the τ.

(3) $$\hat{\Gamma }={\left\{L_{0}^{C_{m}}\right\}}_{m=1}^{n}$$

We also construct a noisy dataset $\phi ={\left\{Nos^{C_{m}}\right\}}_{m=1}^{n}$, and we add this ϕ to the Γ.

(4) $$\Gamma =\hat{\Gamma }+\phi$$

All this sampling method does in the above operation is randomly select data. When we constructed these sub-datasets, we could compute some information between them as new features. Here we define some definitions. First we define TBSS function(transfer by subspace similarity).

**Definition** **1.** *Function $TBSS_{train}$ is defined to generate a new training dataset $T_{1}$ from $T_{0}$ with the setting of $z_{\Gamma }$ (the length of $L_{0}^{C_{m}}$ ) and $z_{\Delta }$ (the length of $ST_{0}^{C_{m}}$ ).*

$$TBSS_{t}rain:SP\times IR\times IR\to SP^{\prime }$$

$$TBSS_{t}rain\left(\tau ,z_{\Delta },z_{\Gamma }\right)={\hat{D}}_{train}$$

*The new table is combined as follows, and the ${\hat{x}}_{e}$ is new instance for a new table.*

$$T_{1}=\left\{{\hat{x}}_{1},{\hat{x}}_{1},\ldots ,{\hat{x}}_{e}\right\}$$

*The features of instance in new table is computed by the function TDT.*

$${\hat{x}}_{e}=\left({\left\{{\left[TDT\right]}_{e,m}\right\}}_{m=1}^{n},c_{i}\right)$$

*${\hat{x}}_{e}$ means we construct a new training data in ${\hat{D}}_{train}$. The new attributes are a group of non-isolation rates and class of this instance is $c_{i}$. A group of non-isolation rates is computed by function ITR () for whose input is a combination of $\left(L_{0}^{C_{i}},ST_{0}^{C_{j}}\right)$, 1≤i≤nand1≤j≤n and $z_{\Gamma }<z_{\Delta }$.*

**Definition** **2.** *Construct a train data table $TDT\in [{\left[0,1\right]}^{n\times n}|C^{n\times 1}]$ and the element in the $i_{th}$ row and $j_{th}$ column of TDT is computed by function $ITR(\;\;L_{0}^{C_{i}},ST_{0}^{C_{j}}\;\;)$ and the element in the $k_{th}$ row and the ${\left(n+1\right)}_{th}$ column is $C_{k}$,1≤i≤nand1≤j≤n:*

(5) $$\begin{array}{c} {\left[TDT\right]}_{i,j}=ITR\left(L_{0}^{C_{i}},ST_{0}^{C_{j}}\right)\\ {\left[TDT\right]}_{k,n+1}=C_{k}\;\;\;\;for\;\;\;\;1\leq k\leq n \end{array}$$

Δ and Γ are sub-datasets from the τ, so the Δ and Γ are subspaces of the τ. We use non-anomaly attribute to represent the similarity of two spaces.

**Definition** **3.** *Function ITR() used X as standard case to train an isolation forest which is an algorithms created by Prof. Zhi Hua Zhou [31] for detecting the isolation point and then put the Y sample set(detection case) into the isolation forest model to classify whether there are isolation points or not in the Y. Finally, computing the rate P of data in Y that normally obeys the distribution in X. In other words, this function is to compute the non-isolation rate of Y.*

(6) ITR:SP×SP→[0,1],ITR(Y,X)=P

### 3.3. Reconstruct Test Data Table

Suppose we have a test dataset. We only use the label of test dataset in evaluation process. We extract the features from each instance. The collection of test dataset is $\hat{d}=\left(y_{i}^{j},c_{n}\right),\hat{d}\in {\hat{D}}_{test}$.

We still apply some transfer processes to the $D_{test}$,transforming $\hat{d}=\left(w_{r}^{j},c_{n}\right),where\hat{d}\in {\hat{D}}_{test}$.

A sliding window is used to construct the sub-dataset. The length of the sliding window is *p*. *p* is same as $z_{\Gamma }$. So the original dataset could be transferred to the $w_{z}=\left\{y_{1+z},y_{2+z},\cdots y_{p+z}\right\}$, where *r* is determined by the length of sliding window *p* and length of test dataset.

**Definition** **4.** *Construct a test data table $TDT_{1}\in {\left[0,1\right]}^{r\times n}$:*

(7) $${\left[TDT_{1}\right]}_{t,j}=ITR\left(w_{t},ST_{0}^{C_{j}}\right)$$

*where $ST_{0}^{C_{j}}=Sam_{md}\left(T_{0}^{C_{j}},z_{\Delta }\right)$, 1≤i≤nand1≤t≤T. $w_{t}$ is the $t_{th}$ sliding window, while the element in the $t_{th}$ row and $j_{th}$ column of $TDT_{1}$ is computed by function $ITR(w_{t},ST_{0}^{C_{j}})$.*

With the help of $TDT_{1}$ the final high level function of step two can be defined as follows.

**Definition** **5.** *Function $TBSS_{test}$ is defined for transforming testing dataset $D_{test}$ into new testing dataset ${\hat{D}}_{test}$ which has same categories of attributes as $T_{1}$. But part of computing input changes because we no longer use τ to gain sample set $L_{0}^{C_{i}}$ whose size is $z_{\Gamma }$ but using sliding windows of $D_{test}$ while the class of new testing data is replaced by the $Maj_{w_{t}}$ which is major class of a sliding window.*

$$TBSS_{test}:SP\times SP\times IR\to SP^{\prime \prime }$$

$$TBSS_{test}\left(\tau ,z_{\Delta },p\right)={\hat{D}}_{test}$$

*where*

$${\hat{D}}_{test}=\left\{w_{1},w_{2},\ldots ,w_{r}\right\}$$

*and for 1≤t≤r,*

(8) $$w_{t}=\left({\left\{{\left[TDT_{1}\right]}_{i,j}\right\}}_{j=1}^{n},Maj_{w_{t}}\right)$$

### 3.4. Step 3: Model Learning

After using the pre-processing method to generate the new training dataset $D_{train}$ and new testing dataset $D_{test}$, user could apply different algorithms to make the prediction with the help of $D_{train}$ and $D_{test}$. Then a group of high level equations that represent these processes is defined as follow:

**Definition** **6.** *${\left\{algo_{i}\right\}}_{i=1}^{5}$ is defined for a group of high level equations that use training data set (such as $D_{train}$), testing dataset (such as $D_{test}$) as well as a group of parameters for training the model and testing the model. Finally the performance evaluation index pf is obtained.*

(9) $$\begin{array}{c} algo_{i}:SP^{\prime }\times SP^{\prime \prime }\times Pre\to IR\\ algo_{i}\left(D_{train},D_{test},P\right)=pf \end{array}$$

*where Pre is a collection of all possible specific parameters with respect to the demand of user and P∈Pre, pf is a performance evaluation index which is a combination of several statistical parameters of model such as accuracy, recall and f1−score. In this paper, there are five classical classification algorithms being tested, they are SVM, logistic regression, C4.5, Bayes classifier and KNN.*

**Figure 2:**
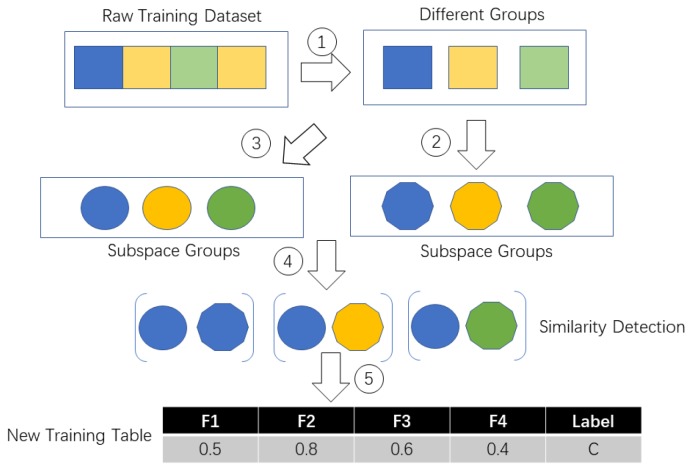
Process of transfering raw training dataset to new training dataset.

**Figure 3:**
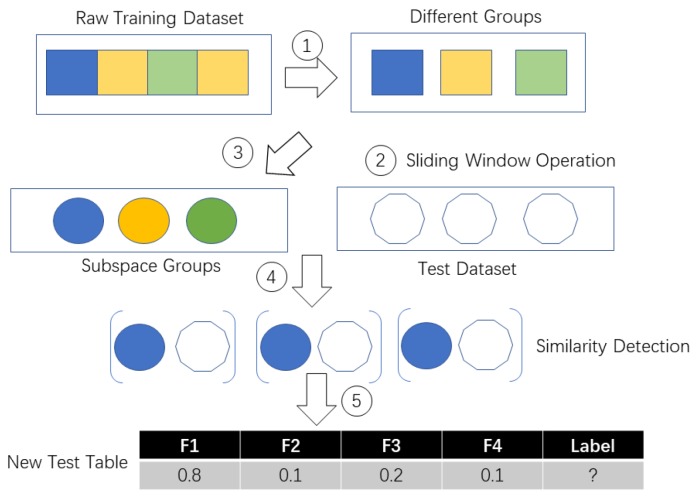
Process of transferring raw test dataset to new test dataset.

In TBSS, the probability between each subspace similarity is calculated. If the subject is walking, of course the data collected under walking status belong to the statistical distribution of walking training dataset. It is known that walking is a homogenous activity without any strange behaviour. So we should obtain a same data space from the walking test data. Time-consumption depends on the time spent on constructing the subspace and the distribution of the original space. If the original space is large (for example, we can possibly have different styles of lying down behavior), the sampling times will be large too because we need to ensure the coverage rate for the original space. If the original space is small (for example we only have a simple kind of walking behavior), the sampling times will be short and the cost time will be low too. Figure 2 and Figure 3 show the TBSS’s process details as well as we introduced the pseudo code of TBSS Algorithm 1 in detail.

After comparing the performance between before and after pre-processing, we found that this new method actually could improve the accuracy of the prediction. The following section will show the experiment results in detail.

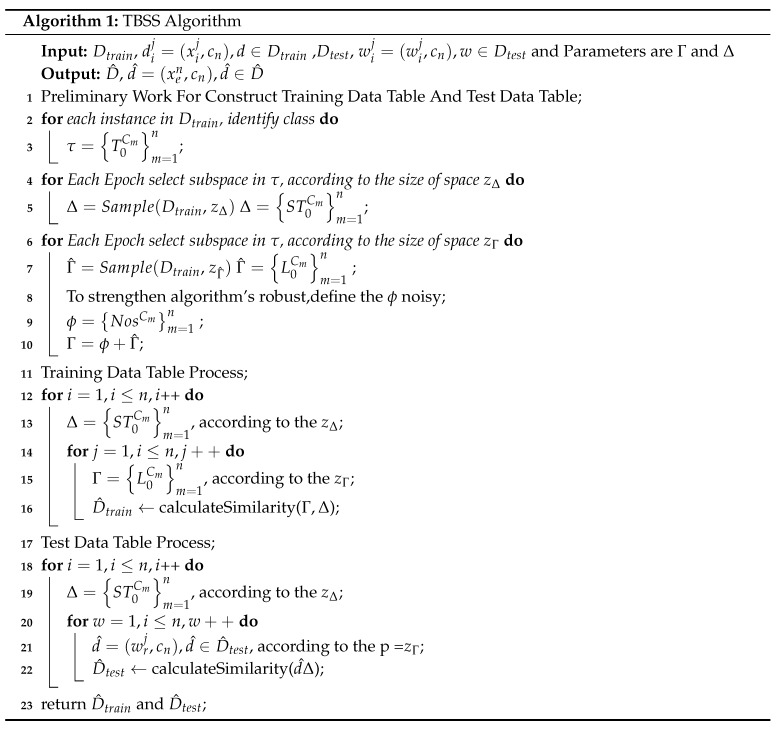



## 4. Experiment

In this section, the performance of the proposed method is evaluated through extensive experiments. We chose three datasets which are generated from some IoT applications, and they are all related to the sensors and ambient assisted environment. The performance of five chosen classification algorithms that function without any pre-processing, serves as a baseline. The baseline is then compared with three pre-processing methods including our new method.

### 4.1. Datasets

**Figure 4:**
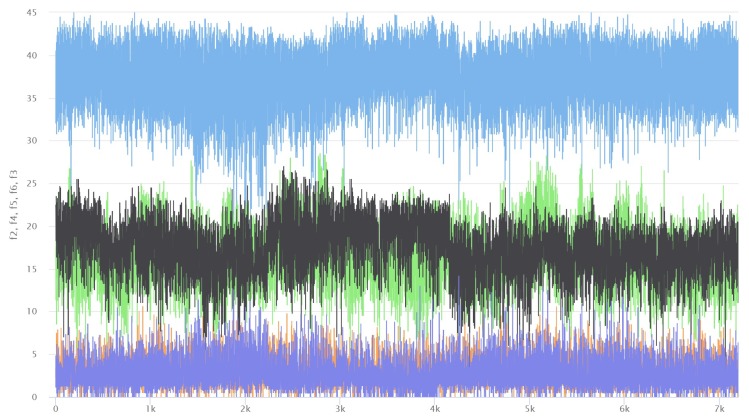
Visualization of AreM dataset which is a graph of time-series of five feature values: it shows that all the time-series are stationary without any significant and distinctive shape, fluctuating within a limited range of values.

Three datasets are prepared in this experiment. All these data are related to the sensor applications, which monitor human activities and environment. The description of each dataset is tabulated in Table 1. These datasets inherently consist of redundancy problem but lack of error information. To simulate the real situation in IoT where faulty sensor can exit, we inject a controlled level of noisy data when we construct the training and testing datasets. As the Figure 4 shows, we analyze some feature of datasets at first. The source datasets are all from the UCI datasets website.

Heterogeneity Activity Recognition Data Set: The Heterogeneity Human Activity Recognition (HHAR) dataset from Smart phones and Smart watches is a dataset devised to benchmark human activity recognition algorithms (classification, automatic data segmentation, sensor fusion, feature extraction, etc.) in real-world contexts; specifically, the dataset is gathered with a variety of different device models and use-scenarios, in order to reflect sensing heterogeneities to be expected in real deployments.

Localization Data for Person Activity Data Set: Data contains recordings of five people performing different activities. Each person wore four sensors (tags) while performing the same scenario five times.

Activity Recognition system based on a Multisensor data fusion (AReM) Data Set: This dataset contains temporal data from a Wireless Sensor Network worn by an actor performing the activities: bending, cycling, lying down, sitting, standing, walking.This dataset represents a real-life benchmark in the area of Activity Recognition applications. The classification tasks consist of predicting the activity performed by the user from time-series generated by a Wireless Sensor Network (WSN), according to the EvAAL competition technical annex

### 4.2. Comparison of Pre-Processing Methods

This section chooses two ways to compare the performance of TBSS. First, five traditional methods were selected as baseline comparisons. There are KNN, Logistic Regression, Naive Bayes, Decision Tree and Support Vector Machine. We try to use the algorithms directly to get the results, then compare with the result that is after TBSS. Second, some pre-processing methods were selected as comparisons.

- K-Neighbors Classifier
- Logistic Regression
- Gaussian Naive Bayes
- Decision Tree
- Support vector machine

Here we select three well-known comparing pre-processing methods as comparisons. They are PCA, Incremental PCA and Normalize Method. Two methods are related to reducing dimension (PCA and Incremental PCA) and one method (Normalize Method) is related to changing the range of the data values. These methods are useful in traditional data mining because they could deal with redundancy problem and dimension explosion.

- PCA
- Incremental PCA
- Normalize Method

### 4.3. Evaluation Criteria

Three evaluation indicators are used for evaluating the pre-processing methods. There are precision, recall and F1-score. These evaluation criteria are very commonly used in classification tasks so we chose these evaluation criteria as a baseline evaluation. In the experiment result, we could focus on the precision criteria. Our proposed methodology performs well in the precision indicator.

- Precision
- Recall
- F1 score

### 4.4. Parameters Setting

For setting the parameters of the comparing methods, default values that are given by the Sklearn package are used. For a fair evaluation of the experiment, the program code is run for ten times and obtain the average precision and recall, etc. The length of the sliding window could be changed to suit different tasks. Further experimentation is planned as future work for exploring the optimal sliding window size. The amount of noise we injected into the original dataset is proportional to the amount of the original dataset.

### 4.5. Result and Analysis

First, we use some original classification algorithms as a baseline to test our datasets. As we could see the results from Table 2, Table 3 and Table 4, the performance of the model that is trained by raw data is not excellent, because the raw data include so much noisy data, and we found that the running time of the whole process is long because the model repeatedly calculated the redundant data.

Table 2 shows that the performance of TBSS and other pre-processing methods on localization recognition dataset. The TBSS pre-processing method is useful to improve the performance of the model. The parameters are set as $z_{\Gamma }=10,z_{\Delta }=20$ and epoch=50. The KNN method does not perform very well in this dataset. Compared with other methods, the TBSS get high precision. Especially the combination model of TBSS and decision tree get the about 0.3264 precision in this dataset.

Table 3 shows that the performance of TBSS and other pre-processing methods on AReM dataset. In this evaluation of dataset, parameters are set as $z_{\Gamma }=10,z_{\Delta }=20$ and epoch=50. As we can see, the TBSS pre-processing is successful in improving the performance of the model by combining with a classification algorithm. The The precision of Bayesian algorithm with TBSS is up to about 0.3477, and it is higher than original precision. Table 4 shows that the performance of TBSS and other pre-processing methods on activity recognition dataset. In this dataset, parameters are set as $z_{\Gamma }=20,z_{\Delta }=30$, epoch=50, and we adjust the interval of data in order to improve higher precision. The TBSS still perform well compared with other methods.

The TBSS gets good results in these datasets, although these datasets have noisy and redundancy problem, because the TBSS focuses on the similarity of space and a subspace, which is not easy to affect by single error instances. We use non-anomaly detection and statistics representing the similarity of each instance. These mechanisms strengthen the model’s robustness and precision.

For a realistic simulation, we design other experiments in which the length of the sliding window ($z_{\Gamma },\;\;z_{\Delta }$) is a variable. When we change $z_{\Gamma },\;\;z_{\Delta }$, the whole model will generates different performance. As Table 5 shows, we fix the value of epoch and set $z_{\Gamma }=10,\;\;z_{\Delta }=20$, $z_{\Gamma }=20,\;\;z_{\Delta }=30$, $z_{\Gamma }=20,\;\;z_{\Delta }=40$, and we use a localization dataset to test the TBSS. It is clear that the setting of $z_{\Gamma }=20,\;\;z_{\Delta }=40$ is better. We analyze this dataset and we found that its classification interval is suitable for the twenty to thirty seconds(which is approximately $z_{\Gamma }=20$). So this result indicated that TBSS methodology is robust with regards to pre-process the sensor data according to the interesting time interval.

The final experiment is to test the influence of epoch. TBSS will receive more instances when the epoch is large because the times of sampling decide the coverage of the subspace to the original space. As Table 6 shows, we set the parameters $z_{\Gamma }=10,\;\;z_{\Delta }=20$, $z_{\Gamma }=20,\;\;z_{\Delta }=30$, $z_{\Gamma }=10,\;\;z_{\Delta }=30$. We try to use 10 to 3 epochs to run the model. We found that the more epochs there are in a range, the greater the improvement in the precision of thenmodel. The epoch does not affect the model’s performance out of a range because the subspace similar is overlapping.

## 5. Conclusions

This paper reports a subspace similarity detection model based on the subspace-attribute probability calculation, and the computational process uses the anomaly detection method. The proposed methodology is to be used as a pre-processing method that transforms the fine granular time scaled dataset (that has frequent intervals) into a probability dataset in a period, hence better classification model training. The TBSS pre-processing method can effectively solve the problem of repeatability and noise that exist in the sensor data. TBSS is able to smoothly combine with anomaly detection and traditional classification algorithm. Therefore it should be flexible for most of the IoT machine learning applications in real life. Through the different aspect of experiments, we observe that this model can effectively improve the performance of traditional machine learning classification algorithms in data mining of the Internet of Things. Although TBSS improves the precision in the sensor data mining task, it has a shortcoming. The time-consumption is relatively long, because the sampling time incurs certain overhead for covering the whole search space in each step of window sliding. In the future work, we will look into formulating a low-complexity design suitable for fast sampling for TBSS. The codes of TBSS should be optimized for leveraging GPU computation for fast running.

## Figures and Tables

**Table 1:** Description of various datasets.

Dataset	Instances	Features	Labels	Noisy
LD	164860	8	8	10000
HHAR	43930257	16	6	100000
AReM	42240	6	4	1000

**Table 2:** The performance of TBSS and other pre-processing methods on localization recognition dataset.

Model	Precision	Recall	F1 Score
Localization Dataset ($z_{\Gamma }=10,z_{\Delta }=20$)			
KNN	0.1721 ± 0.018	0.3015 ± 0.011	0.2134 ± 0.019
KNN+PCA	0.1154 ± 0.016	0.2125 ± 0.013	0.1363 ± 0.014
KNN+Normalize	0.1422 ± 0.012	0.2614 ± 0.015	0.1832 ± 0.009
KNN+IPCA	0.0134 ± 0.017	0.1683 ± 0.029	0.1035 ± 0.027
KNN+TBSS	0.0467 ± 0.028	0.0454 ± 0.014	0.0335 ± 0.018
DT	0.1811 ± 0.015	0.2436 ± 0.025	0.2021 ± 0.019
DT+PCA	0.1043 ± 0.018	0.1653 ± 0.029	0.1137 ± 0.025
DT+Normalize	0.1457 ± 0.028	0.2134 ± 0.014	0.1612 ± 0.018
DT+IPCA	0.0854 ± 0.035	0.1210 ± 0.023	0.0814 ± 0.037
DT+TBSS	0.3264 ± 0.016	0.0978 ± 0.015	0.1248 ± 0.026
SVM	0.1321 ± 0.035	0.3014 ± 0.029	0.1731 ± 0.013
SVM+PCA	0.0934 ± 0.019	0.2426 ± 0.028	0.1367 ± 0.018
SVM+Normalize	0.0823 ± 0.031	0.2464 ± 0.012	0.1134 ± 0.019
SVM+IPCA	0.1012 ± 0.027	0.2810 ± 0.036	0.1521 ± 0.021
SVM+TBSS	0.1634 ± 0.022	0.0767 ± 0.010	0.0936 ± 0.019
LG	0.1311 ± 0.029	0.3023 ± 0.019	0.1763 ± 0.016
LG+PCA	0.0325 ± 0.026	0.0312 ± 0.028	0.0853 ± 0.019
LG+Normalize	0.0821 ± 0.009	0.2516 ± 0.008	0.1274 ± 0.007
LG+IPCA	0.1234 ± 0.020	0.2346 ± 0.010	0.1474 ± 0.012
LG+TBSS	0.2668 ± 0.025	0.1121 ± 0.019	0.1453 ± 0.016
NB	0.1333 ± 0.022	0.2745 ± 0.018	0.1624 ± 0.017
NB+PCA	0.0929 ± 0.014	0.2535 ± 0.018	0.1374 ± 0.019
NB+Normalize	0.1153 ± 0.013	0.2074 ± 0.018	0.1136 ± 0.018
NB+IPCA	0.1167 ± 0.012	0.2963 ± 0.011	0.1526 ± 0.019
NB+TBSS	0.2442 ± 0.011	0.0947 ± 0.013	0.1163 ± 0.029

**Table 3:** The performance of TBSS and other pre-processing methods on AReM recognition dataset.

Model	Precision	Recall	F1 Score
AReM Dataset ($z_{\Gamma }=10,z_{\Delta }=20$)			
KNN	0.1743 ± 0.016	0.1945 ± 0.014	0.1324 ± 0.012
KNN+PCA	0.3053 ± 0.016	0.2631 ± 0.018	0.2734 ± 0.016
KNN+Normalize	0.1784 ± 0.014	0.1982 ± 0.028	0.1335 ± 0.014
KNN+IPCA	0.3033 ± 0.101	0.2613 ± 0.018	0.2743 ± 0.026
KNN+TBSS	0.3042 ± 0.026	0.2634 ± 0.025	0.2733 ± 0.018
DT	0.1832 ± 0.010	0.2421 ± 0.015	0.2053 ± 0.015
DT+PCA	0.3453 ± 0.013	0.2442 ± 0.018	0.2042 ± 0.016
DT+Normalize	0.4935 ± 0.024	0.2674 ± 0.032	0.2434 ± 0.018
DT+IPCA	0.1845 ± 0.012	0.1534 ± 0.012	0.1057 ± 0.014
DT+TBSS	0.3037 ± 0.019	0.3353 ± 0.018	0.3073 ± 0.015
SVM	0.1335 ± 0.012	0.3073 ± 0.022	0.1753 ± 0.017
SVM+PCA	0.2675 ± 0.010	0.2443 ± 0.018	0.2123 ± 0.016
SVM+Normalize	0.3554 ± 0.017	0.3943 ± 0.012	0.2532 ± 0.012
SVM+IPCA	0.2642 ± 0.010	0.2421 ± 0.018	0.2122 ± 0.016
SVM+TBSS	0.2844 ± 0.013	0.2142 ± 0.013	0.2242 ± 0.009
LG	0.1334 ± 0.013	0.3034 ± 0.009	0.1732 ± 0.016
LG+PCA	0.2586 ± 0.010	0.2394 ± 0.028	0.1975 ± 0.018
LG+Normalize	0.2832 ± 0.020	0.2212 ± 0.042	0.2523 ± 0.032
LG+IPCA	0.2212 ± 0.010	0.1863 ± 0.038	0.1524 ± 0.036
LG+TBSS	0.3234 ± 0.029	0.2413 ± 0.014	0.2523 ± 0.021
NB	0.1313 ± 0.022	0.2726 ± 0.025	0.1613 ± 0.027
NB+PCA	0.3086 ± 0.010	0.2426 ± 0.018	0.2111 ± 0.016
NB+Normalize	0.3132 ± 0.023	0.1713 ± 0.009	0.0239 ± 0.010
NB+IPCA	0.2845 ± 0.015	0.2234 ± 0.027	0.2132 ± 0.023
NB+TBSS	0.3477 ± 0.020	0.2663 ± 0.017	0.1856 ± 0.012

**Table 4:** The performance of TBSS and other pre-processing methods on activity recognition dataset.

Model	Precision	Recall	F1 Score
Activity Recognition ($z_{\Gamma }=20,z_{\Delta }=30$)			
KNN	0.1734 ± 0.027	0.3023 ± 0.011	0.2113 ± 0.019
KNN+PCA	0.1543 ± 0.016	0.1434 ± 0.014	0.1665 ± 0.025
KNN+Normalize	0.1932 ± 0.028	0.2075 ± 0.024	0.2157 ± 0.023
KNN+IPCA	0.1374 ± 0.016	0.1834 ± 0.014	0.1656 ± 0.015
KNN+TBSS	0.0934 ± 0.015	0.1074 ± 0.013	0.0455 ± 0.013
DT	0.2664 ± 0.014	0.2767 ± 0.015	0.2568 ± 0.014
DT+PCA	0.2964 ± 0.016	0.2221 ± 0.018	0.2468 ± 0.025
DT+Normalize	0.2635 ± 0.013	0.2867 ± 0.013	0.2434 ± 0.024
DT+IPCA	0.3168 ± 0.024	0.2878 ± 0.023	0.2767 ± 0.027
DT+TBSS	0.3441 ± 0.027	0.2342 ± 0.039	0.3084 ± 0.024
SVM	0.1345 ± 0.022	0.3066 ± 0.012	0.1753 ± 0.037
SVM+PCA	0.3105 ± 0.026	0.2368 ± 0.046	0.2323 ± 0.013
SVM+Normalize	0.3243 ± 0.023	0.2163 ± 0.035	0.2435 ± 0.033
SVM+IPCA	0.2532 ± 0.016	0.2463 ± 0.046	0.2513 ± 0.014
SVM+TBSS	0.3021 ± 0.035	0.2147 ± 0.033	0.2373 ± 0.013
LG	0.1924 ± 0.013	0.2432 ± 0.010	0.1703 ± 0.010
LG+PCA	0.2523 ± 0.029	0.2154 ± 0.024	0.2163 ± 0.014
LG+Normalize	0.2935 ± 0.026	0.1964 ± 0.016	0.2243 ± 0.035
LG+IPCA	0.2626 ± 0.037	0.2172 ± 0.013	0.2025 ± 0.015
LG+TBSS	0.2834 ± 0.016	0.2053 ± 0.014	0.2153 ± 0.015
NB	0.2653 ± 0.024	0.2753 ± 0.025	0.3026 ± 0.014
NB+PCA	0.2923 ± 0.014	0.2845 ± 0.015	0.2624 ± 0.053
NB+Normalize	0.3129 ± 0.012	0.2965 ± 0.023	0.3024 ± 0.012
NB+IPCA	0.3123 ± 0.023	0.2754 ± 0.023	0.2636 ± 0.014
NB+TBSS	0.3534 ± 0.017	0.2623 ± 0.034	0.2753 ± 0.027

**Table 5:** Different size of subspace and its performance.

Localization Dataset			
**Model (TBSS)**	**Precision**	**Recall**	**F1 Score**
$z_{\Gamma }=10,\;\;z_{\Delta }$ = 20			
KNN	0.0612 ± 0.012	0.0723 ± 0.011	0.0353 ± 0.012
DT	0.3134 ± 0.032	0.0713 ± 0.017	0.1462 ± 0.016
SVM	0.2052 ± 0.013	0.0746 ± 0.013	0.1134 ± 0.032
LG	0.2852 ± 0.038	0.1153 ± 0.013	0.1524 ± 0.013
NB	0.2626 ± 0.011	0.1163 ± 0.013	0.1323 ± 0.012
$z_{\Gamma }=20,\;\;z_{\Delta }$ = 30			
KNN	0.0253 ± 0.010	0.0623 ± 0.011	0.0352 ± 0.011
DT	0.2353 ± 0.022	0.1275 ± 0.041	0.1253 ± 0.023
SVM	0.3125 ± 0.012	0.1035 ± 0.012	0.1153 ± 0.012
LG	0.1834 ± 0.042	0.0734 ± 0.014	0.0835 ± 0.014
NB	0.1953 ± 0.031	0.0922 ± 0.021	0.1073 ± 0.011
$z_{\Gamma }=20,\;\;z_{\Delta }$ = 40			
KNN	0.0134 ± 0.007	0.0324 ± 0.008	0.0132 ± 0.005
DT	0.2035 ± 0.056	0.1652 ± 0.022	0.1764 ± 0.012
SVM	0.4212 ± 0.010	0.1662 ± 0.024	0.1823 ± 0.032
LG	0.1626 ± 0.033	0.1023 ± 0.012	0.1043 ± 0.042
NB	0.3017 ± 0.028	0.0845 ± 0.012	0.1115 ± 0.051

**Table 6:** Different iteration times to the performance of the classification in LG dataset.

Parameters	Epoch	Precision	Recall	F1 score
$z_{\Gamma }=10,\;\;z_{\Delta }=20$	10	0.2952 ± 0.025	0.0942 ± 0.012	0.1343 ± 0.022
	20	0.3045 ± 0.032	0.1084 ± 0.032	0.1253 ± 0.022
	30	0.3264 ± 0.023	0.1311 ± 0.023	0.1442 ± 0.014
	40	0.3323 ± 0.022	0.1434 ± 0.013	0.1503 ± 0.035
	50	0.3353 ± 0.015	0.1334 ± 0.024	0.1452 ± 0.024
$z_{\Gamma }=20,\;\;z_{\Delta }=30$	10	0.3035 ± 0.010	0.0823 ± 0.013	0.1224 ± 0.022
	20	0.3130 ± 0.012	0.1026 ± 0.012	0.1164 ± 0.009
	30	0.3364 ± 0.024	0.1282 ± 0.034	0.1033 ± 0.013
	40	0.3464 ± 0.013	0.1374 ± 0.024	0.1079 ± 0.031
	50	0.3457 ± 0.022	0.1275 ± 0.015	0.1135 ± 0.023
$z_{\Gamma }=10,\;\;z_{\Delta }=30$	10	0.4223 ± 0.010	0.1452 ± 0.025	0.1653 ± 0.024
	20	0.4232 ± 0.030	0.1674 ± 0.024	0.1854 ± 0.022
	30	0.4423 ± 0.012	0.1534 ± 0.016	0.1734 ± 0.033
	40	0.4564 ± 0.020	0.1241 ± 0.015	0.1477 ± 0.032
	50	0.4542 ± 0.028	0.1537 ± 0.025	0.1652 ± 0.025
